# Neuroprotective Effects of Radix Scrophulariae on Cerebral Ischemia and Reperfusion Injury via MAPK Pathways

**DOI:** 10.3390/molecules23092401

**Published:** 2018-09-19

**Authors:** Xiangbao Meng, Weijie Xie, Quanfu Xu, Tian Liang, Xudong Xu, Guibo Sun, Xiaobo Sun

**Affiliations:** 1 Beijing Key Laboratory of Innovative Drug Discovery of Traditional Chinese Medicine (Natural Medicine) and Translational Medicine, Institute of Medicinal Plant Development, Peking Union Medical College and Chinese Academy of Medical Sciences, Beijing 100193, China; xbmeng@implad.ac.cn (X.M.); ginseng123@163.com (W.X.); shengjupan@163.com (Q.X.); liantianparper@sina.com (T.L.); 2 Key Laboratory of Bioactive Substances and Resource Utilization of Chinese Herbal Medicine, Ministry of Education, Beijing 100193, China; 3 Key Laboratory of Efficacy Evaluation of Chinese Medicine against Glycolipid Metabolic Disorders, State Administration of Traditional Chinese Medicine, Beijing 100193, China; 4 Zhongguancun Open Laboratory of the Research and Development of Natural Medicine and Health Products, Beijing 100193, China

**Keywords:** Radix Scrophulariae, cerebral ischemia stroke, middle cerebral artery occlusion-reperfusion, apoptosis, oxygen-glucose deprivation and reperfusion, MAPK pathways

## Abstract

Ischemic stroke is a clinically common cerebrovascular disease whose main risks include necrosis, apoptosis and cerebral infarction, all caused by cerebral ischemia and reperfusion (I/R). Ischemia and reperfusion-induced injury or apoptosis inhibition in human brain tissue may exert an irreplaceable protective effect on ischemic nerves. This process has particular significance for the treatment of stroke patients. However, the development of neuroprotective drugs remains challenging. Radix Scrophulariae, traditionally considered a valuable medicine, has been discovered to have neuroprotective effects. To explore the neuroprotective effects of an aqueous extract of Radix Scrophulariae (RSAE) on cerebral ischemia/reperfusion and their underlying mechanisms, oxygen-glucose deprivation and reperfusion (OGD/R)-induced PC12 cells were used, and a middle cerebral artery occlusion/reperfusion (MCAO/R) mouse model was established. In vitro results showed that 12.5 μg/mL RSAE markedly improved cell viability; inhibited LDH leakage; increased SOD, GSH-Px and CAT enzyme activity; stabilized the mitochondrial membrane potential; and reduced OGD-induced cell injury and apoptosis. Additionally, in vivo results preliminarily suggested that in MCAO/R model mice, RSAE treatments attenuated infarct volume; reduced brain water content and nitric oxide (NO) and malondialdehyde (MDA) concentrations; inhibited I/R-induced neurological deficits; reduced the levels of lactate dehydrogenase (LDH) leakage release; improved antioxidant capacity by upregulating SOD, GSH-Px and CAT enzyme activity; and reduced neuronal apoptosis, necrosis and loss of neurons. Moreover, it was found that RSAE upregulated the expression of Bcl-2 and downregulated the expression of Bax. In addition, the phosphorylation levels of MAPK signal pathways were elucidated via western blot analysis and immunohistochemical evaluation. In summary, this study investigated the neuroprotective effects and potential mechanisms of RSAE on focal cerebral I/R injury in mice. Radix Scrophulariae has been previously identified as a potential neuroprotective natural plant. Hence, our results may offer insight into discovering new active compounds or drugs for the treatment of ischemic stroke. Many new natural active chemicals in this extract may be discovered by chemical separation and identification and may provide new insights into therapeutic targets in stroke patients.

## 1. Introduction

Ischemic stroke is a clinically common cerebrovascular disease accounting for 70 to 80% of all cerebrovascular patients; approximately 15 million people worldwide suffer from stroke each year, resulting in approximately 5 million deaths and making it a primary cause of disability and death worldwide [1,2]. Thus far, the primary concern of clinical treatment for ischemic stroke is to restore blood and oxygen supply to the ischemic brain tissue as soon as possible. However, re-opening the occluded cerebral vessels often produces pathological damage in the ischemic tissue and causes cerebral ischemia and reperfusion injury to be aggravated further or potentially rendered irreversible [2,3]. To explore better treatment options for ischemic stroke and reperfusion injury, researchers have carried out extensive studies on its pathogenesis; proposed a set of theories [4,5] mainly including energy metabolism disorders, oxidative stress, glutamate toxicity, Ca^2+^ overload, excessive NO synthesis, and apoptosis; and developing a series of new drugs [5], such as recombinant tissue plasminogen activator (r-TPA), aspirin and heparin. At present, significant progress has been made to alleviate the tremendous public health problems relating to stroke, such as the preventive efforts to reduce the morbidity and mortality associated with strokes, in addition to the establishment of special intensive care units to improve the functional consequence of stroke patients. However, the management of acute ischemic stroke has not made significant strides since the introduction of recombinant tissue plasminogen activator (r-TPA) two decades ago [3], and r-TPA is the only thrombolytic agent approved by the US Food and Drug Administration for stroke treatment [6]. Since its therapeutic time window (TTW) is narrow, r-TPA treatment for stroke is suitable for only a limited percentage of stroke patients (<10%) [7].

After cerebral ischemia, particularly following the cerebral ischemia and reperfusion process, various damage factors exist, including oxygen free radicals, oxidative stress, glutamate toxicity, Ca^2+^ overload, and excessive NO synthesis. Furthermore, apoptosis is triggered through various pathways, such as hydroxyl radicals and superoxide anions. In the pathogenesis and treatment of acute ischemic stroke, an acute ischemic cascade reaction can greatly damage brain cells due to energy metabolism disorders, oxidative stress or inhibition of the body’s oxidative defence, easily leading to neuronal apoptosis, necrosis or loss of neurons in the hippocampus and cortex of the brain [8,9,10,11,12,13]. Therefore, research and development of neuroprotective drugs to reduce apoptosis and necrosis of brain tissue for the treatment of acute ischemic stroke is clinically imperative [14].

Apoptosis, also called programmed cell death, is involved in the pathogenesis and pathological mechanism of ischemic stroke. Apoptosis is widely involved in the activation of cell development and physiological cell turnover but can also contribute to pathological damage by environmental stimulation, leading to disorders such as stroke [12,14,15,16,17]. It has been shown that arsenic-induced cytotoxicity and oxidative stress damage could lead to apoptosis in a variety of cell types, including neuronal cells, myoblasts, and osteoblasts, which are closely related to activation of PI3K-Akt/mTOR, mitogen-activated protein kinase (MAPK) and endoplasmic reticulum stress regulatory signaling pathways [3,4,8,9,10]. While the mechanisms of neuronal apoptosis caused by cerebral ischemia are not yet completely elucidated, much evidence has shown that neuronal apoptosis is closely associated with cell cycle regulation and oxidative stress damage. Furthermore, the MAPK signaling pathway, which includes ERK1/2, JNK1/2 and p-38, may inhibit the activation and hyperproliferation of glial cells by regulating the cell cycle and reducing harmful secreted factors that damage neurons [8,9,10,13]; on the other hand, this pathway reduces glial proliferation and swelling caused by local microcirculatory disorders, which creates a favorable environment for survival, not neuronal apoptosis [18]. The MAPK signaling pathway has been implicated in the regulation of cytokine expression and cell apoptosis after stroke, and this pathway might represent a novel therapeutic target [19,20]. Thus, studies of MAPK activation in ischemic brain tissue may provide a foundation for the discovery of novel therapeutic agents for stroke patients.

In China, the use of traditional Chinese herbal medicines and combination preparations to treat cerebrovascular diseases can be traced back to the Han Dynasty. Even thousands of years ago, the medical formulary recorded many valuable Chinese medicines and classic prescriptions, such as melon, salvia, mulberry, panax notoginseng, and ganoderma lucidum, for the treatment of brain I/R-related diseases [21]. Recently, several new compound traditional Chinese medicine preparations have been formulated for the treatment of cerebrovascular diseases in China [15,16,21]. Radix Scrophulariae, called Xuan Shen in Chinese, is a traditional Chinese medicine derived from the dried roots of *Scrophularia ningpoensis* Hemsl. Radix Scrophulariae has been used for thousands of years in China owing to its excellent traditional therapeutic effects and various pharmacological activities, such as anti-myocardial ischemia, anti-atherosclerosis, antimyocardial hypertrophy, anticerebral ischemia, antiplatelet aggregation, anti-inflammatory, liver protection, immune regulation, antibacteria, neuron protection, hypnosis, and antihyperuricaemia [22,23]. Modern pharmacological studies and clinical practice have demonstrated that Radix Scrophulariae possesses anti-angiogenesis, anti-inflammatory, and antimicrobial activities, as well as the ability to promote ventricular remodeling [21,22,24]. Some studies have demonstrated that Radix Scrophulariae extract has anti-apoptotic and anti-inflammatory effects, potentially operating by affecting the mitogen-activated protein kinases (MAPKs) signaling pathway and inhibition of the NF-κB pathway [24]. Although some pharmacological studies have been reported, the effects and mechanisms of Radix Scrophulariae activity against cerebral ischemia has not been clearly elaborated, and the chemical basis of this activity remains unclear.

**Figure 1:**
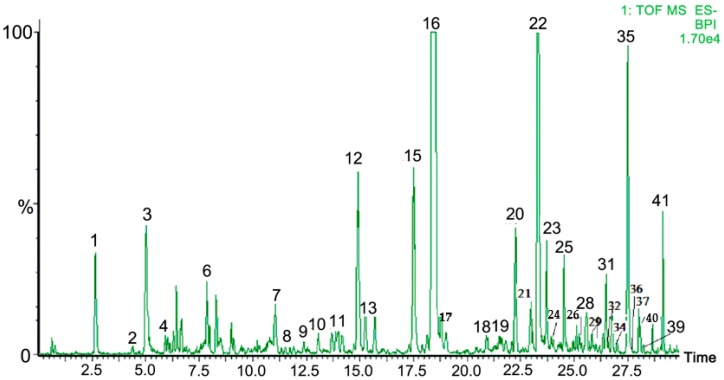
Total ion chromatograms (TIC) of Radix Scrophulariae aqueous extract in the negative ion mode via UPLC-QTOF-MS/MS.

Based on the above, we hypothesized that Radix Scrophulariae extract may provide neuroprotective effects on cerebral ischemia and reperfusion injury by inhibiting oxidative stress damage and inhibiting apoptosis by regulating the MAPK pathway. Therefore, we investigated the effects of Radix Scrophulariae aqueous extract (RSAE) on PC12 cells subjected to an oxygen-glucose deprivation and reperfusion (OGD/R) model and MCAO/R-operated mice in cerebral ischemic stroke. Recently, chemists have identified various chemical components within this extract, such as iridoids, phenylpropanoids, anthraquinones, phenols, phenylethanoid glycosides, sterols, flavonoids, fatty acids, and sugars; as showed in Figure 1, a total of 41 compounds were identified from RSAE based on the accurate mass measurement of precursor and product ions via MS/MS-Q-TOF/MS as previously described [22,23].

## 2. Results

### 2.1. Cell Viability in PC12 Cells after OGD/R

**Figure 2:**
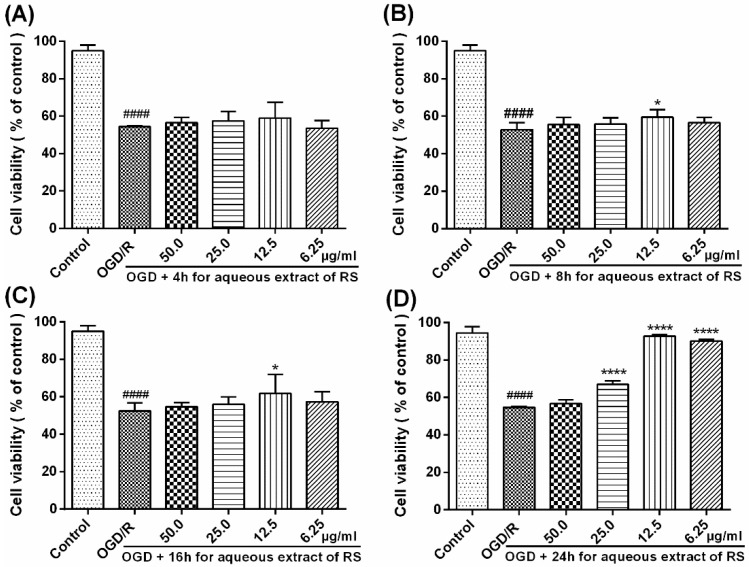
Effects of Radix Scrophulariae aqueous extract on the viability of PC12 cells after OGD/R. After PC12 cells were exposed to 2 h of OGD following 24 h of reperfusion, cell viability was significantly decreased. (**A**) Incubation of PC12 cells with different concentrations of RSAE (5.0, 12.5, 25.0 and 50.0 μg/mL) alone for 4 h did not affect cell viability. (**B**,**C**) Pretreatment with RSAE (12.5 μg/mL) for 8 h and 16 h significantly improved cell viability. (**D**) After PC12 cells were exposed to 2 h of OGD following 24 h of reperfusion, pretreatment with RSAE (5.0, 12.5, 25.0 and 50.0 μg/mL) for 24 h significantly improved cell viability. Mean values ± standard error of the mean (n = 6); * *p* < 0.05, **** *p* < 0.00001 versus OGD/R group; ^#^ *p* < 0.05, ^##^ *p* < 0.01, ^####^ *p* < 0.0001 versus control group.

**Figure 3:**
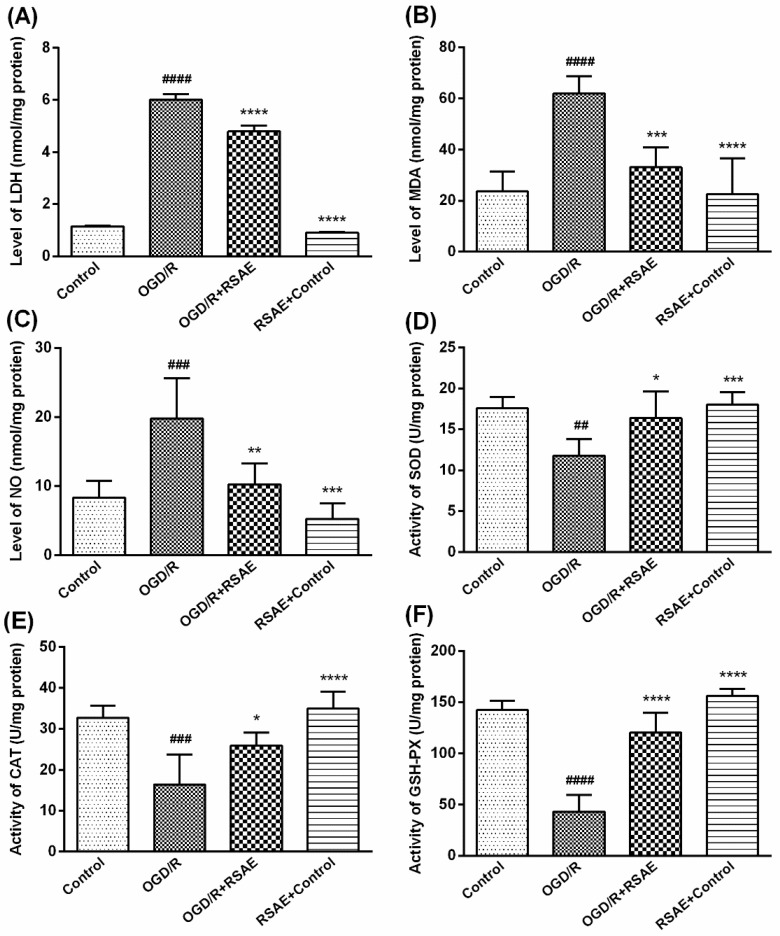
Effects of RSAE on the levels of LDH leakage, MDA and oxidative stress in PC12 cells after OGD/R. A concentration of 12.5 μg/mL RSAE inhibited OGD/R-induced damage and decreased the levels of LDH leakage (**A**), MDA (**B**) and NO (**C**). Pretreatment with RSAE (12.5 μg/mL) for 24 h significantly improved SOD (**D**), CAT (**E**) and GSH-Px (**F**) activity. Mean values ± standard error of the mean (n = 6); * *p* < 0.05, *** *p* < 0.001, **** *p* < 0.0001 versus OGD/R group; ^#^ *p* <0.05, ^##^ *p* < 0.01, ^####^ *p* < 0.0001 versus control group.

The neuronal viability of PC12 pheochromocytoma cells was assessed by the MTT assay, and cell damage was evaluated by measuring the amount of extracellular LDH leakage release. As shown in Figure 2, 2 h of OGD followed by 24 h of reperfusion caused a significant decrease (*p* < 0.0001) in the viability of PC12 cells, while the level of LDH leakage release was remarkably increased (*p* < 0.0001, Figure 3A). By contrast, incubation of cells with varying concentrations of Radix Scrophulariae aqueous extract (RSAE: 6.25, 12.5, 25.0 and 50.0 μg/mL) alone for 4 h did not affect cell viability (Figure 2A). Pretreatment of PC12 cells with different concentrations of RSAE for 24 h significantly increased cell viability from 60.11% to 66.86%, 69.07%, 92.42%, and 83.21% (*p* < 0.0001, Figure 2D) in a dose-dependent manner. Furthermore, the 12.5 μg/mL RSAE treatment had a much greater effect on improving cell viability than the other doses. Therefore, the 12.5 μg/mL RSAE concentration was selected for use in subsequent experiments. Moreover, the results indicated that higher concentrations of RSAE actually had weaker effects than lower concentrations. Thus, RSAE may have some toxic effects at higher concentrations (greater than 50.0 μg/mL).

### 2.2. Levels of LDH Leakage, MDA, and Oxidative Stress in PC12 Cells after OGD/R

As shown in Figure 3, after PC12 cells were exposed to the oxygen-glucose deprivation/reperfusion (OGD/R) treatment, the level of LDH leakage release remarkably increased (*p* < 0.0001, Figure 3A) compared with that of the control group, but the levels of LDH leakage release were markedly reduced by 12.5 μg/mL Radix Scrophulariae aqueous extract (RSAE), with levels decreasing from 6.05 ± 0.16 to 4.8 ± 0.26 (*p* < 0.0001, Figure 3A).

Compared with control group, OGD/R treatment markedly increased the concentrations of MDA from 25.84 ± 7.09 to 64.81 ± 2.23 and those of NO from 8.36 ± 2.75 to 19.79 ± 6.71 (*p* < 0.001, Figure 3B,C). However, 12.5 μg/mL RSAE inhibited OGD/R-induced damage and decreased the concentrations of MDA and NO to 34.24 ± 8.36 and 10.24 ± 3.52, respectively (*p* < 0.001, Figure 3B,C).

In addition, after PC12 cells were exposed to OGD/R treatment, the activity of SOD, CAT and GSH-Px remarkably decreased compared to that in the control groups, as shown in Figure 3D (from 17.60 ± 1.03 to 11.79 ± 2.25, *p* < 0.01), Figure 3E (from 32.71 ± 2.29 to 16.39 ± 8.20, *p* < 0.001) and Figure 3F (from 142.38 ± 9.04 to 43.05 ± 16.46, *p* < 0.0001), respectively. However, 12.5 μg/mL RSAE pretreatment significantly increased SOD, GSH-Px and CAT activity in OGD/R-treated PC12 cells, as displayed in Figure 3D (from 11.79 ± 2.25 to 16.40 ± 2.96, *p* < 0.05), E (from 16.39 ± 8.20 to 25.94 ± 3.61, *p* < 0.05) and F (from 43.05 ± 16.46 to 120.42 ± 19.09, *p* < 0.001), respectively.

### 2.3. Mitochondrial Membrane Potential in PC12 Cells after OGD/R

**Figure 4:**
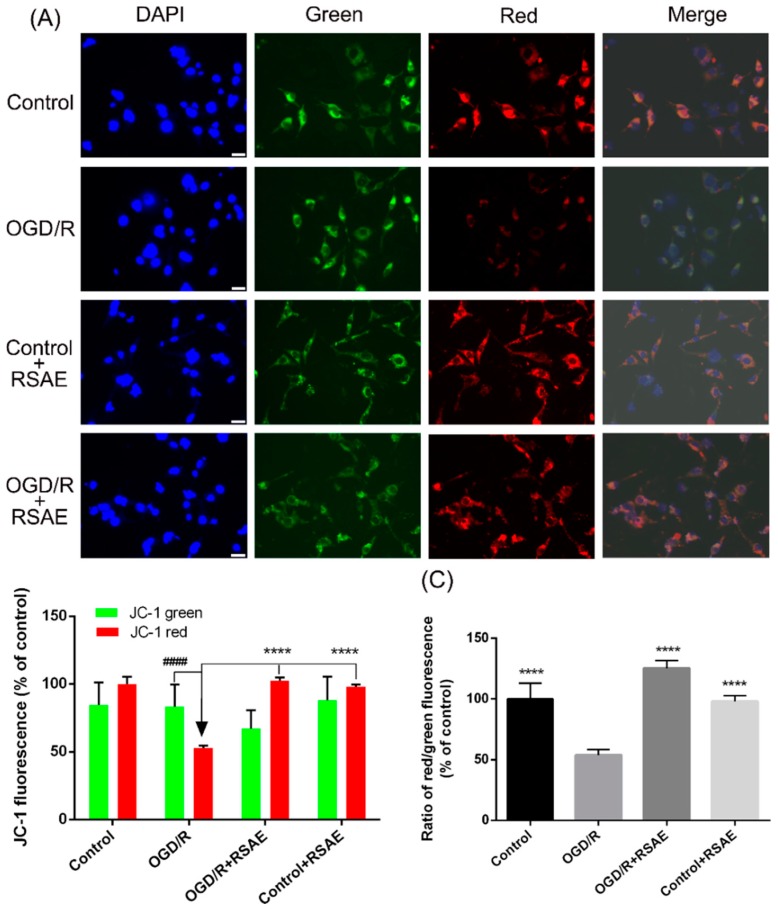
Effects of RSAE on the mitochondrial membrane potential in PC12 cells after OGD/R. Effects of RSAE on the MMP in PC12 cells after OGD/R. A, concentration of 12.5 μg/mL RSAE inhibited the OGD/R-induced reduction in the MMP (**A**,**B**) and increased the ratio of red/green fluorescence intensity (**C**). Mean values ± standard error of the mean (n = 6); **** *p* < 0.0001 versus OGD/R group; ^#^ *p* < 0.05, ^##^ *p* < 0.01, ^####^ *p* < 0.0001 versus control group. Scale bar, 20 μm.

The reduction in the mitochondrial membrane potential (MMP) is linked with OGD/R-induced apoptosis and is regarded as a vital event during apoptosis. Therefore, JC-1 staining was used to detect changes in the MMP. Figure 4A,B showed that compared with the control group, OGD/R markedly weakened the red fluorescence intensity from 1.00 ± 0.13 to 0.52 ± 0.043 (*p* < 0.001), while it caused a slight fluctuation in the green fluorescence intensity among these groups. However, 12.5 μg/mL RSAE inhibited the reduction in the MMP induced by OGD/R, increasing the red fluorescence to 1.02 ± 0.05 (*p* < 0.001, Figure 4B) and decreasing the green fluorescence from 0.98 ± 0.052 to 0.82 ± 0.45. The ratio of red/green fluorescence intensity was greatly enhanced by RSAE, increasing from 0.52 ± 0.043 to 1.25 ± 0.065 (*p* < 0.001, Figure 4C), which was consistent with the red fluorescence result. Furthermore, RSAE and nimodipine are similar in their enhancement of red fluorescence (*p* < 0.0001, Figure 4B).

### 2.4. Infarct Volume, Brain Water Content, MDA, NO Concentrations, and SOD Activity

**Figure 5:**
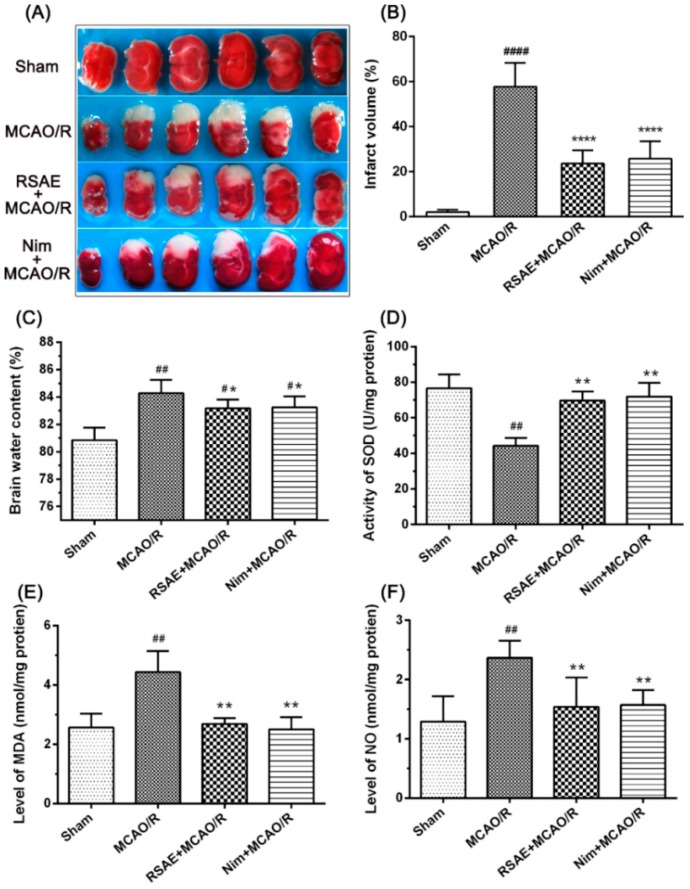
Effects of RSAE on infarct volume, brain water content, MDA, and NO concentrations and SOD activity in mice exposed to MCAO/R injury. RSAE reduced the infarct volume and brain water content (BWC). (**A**) Representative images of TTC-stained brain sections from the sham-operated or RSAE-treated animals collected 24 h after infarction. (**B**) Quantitative analysis of the infarct volume. (**C**) Brain water content in ischemia hemispheres of all groups. (**D**) The activity of SOD in ischemic hemispheres of all groups. (**E**) The concentration of MDA in ischemic hemispheres of all groups. (**F**) The concentration of NO in ischemic hemispheres of all groups. Mean values ± standard error of the mean (n = 10); * *p* < 0.05, ** *p* < 0.01, **** *p* < 0.0001 versus MCAO/R group; ^#^ *p* < 0.05, ^##^ *p* < 0.01, ^####^ *p* < 0.0001 versus sham-operated group.

To assess the effects of RSAE on focal brain ischemic injury, KM mice were subjected to 2 h of MCAO and 24 h of reperfusion. The infarct volume of the ipsilateral brain was measured with TTC staining (Figure 5A). As shown in Figure 5A,B, the MCAO/R operation significantly increased the corrected infarct volumes compared to those in the sham operation group (*p* < 0.0001, Figure 5A,B). However, pretreatment with RSAE (2.4 g·kg^−1^ of the original drug) and 12 mg·kg^−1^ of nimodipine for 7 d, resulted in a remarkable decrease in the corrected infarct volumes compared to those in the MCAO/R group (*p* < 0.0001, Figure 5A,B).

To evaluate brain edema in the ischemic brain region, we examined the brain water content of all the groups. The results showed that the brain water content (BWC) distinctly increased in the ischemic brain hemisphere of the MCAO/R group, and this increase was significantly reduced by pretreatment with RSAE (2.4 g·kg^−1^ of the original drug) (*p* < 0.0001, Figure 5C) and 12 mg·kg^−1^ nimodipine (*p* < 0.0001, Figure 5C). RSAE and nimodipine are similar in their ability to reduce brain water content (*p* < 0.0001, Figure 5C).

Moreover, compared with the sham operation, the MCAO/R operation obviously reduced the activity of SOD from 76.61 ± 7.67 to 44.38 ± 5.91 (*p* < 0.01, Figure 5D) and increased the concentration of MDA from 2.56 ± 0.47 to 4.43 ± 0.99 (*p* < 0.01, Figure 5E) and that of NO from 1.29 ± 0.43 to 2.37 ± 0.40 (*p* < 0.01, Figure 5F). However, pretreatment with RSAE inhibited the MCAO/R-induced ischemic damage and increased the activity of SOD to 69.63 ± 7.14 (*p* < 0.01, Figure 5D). The concentrations of MDA and NO decreased to 2.68 ± 0.27 and 1.54 ± 0.69, respectively (*p* < 0.01, Figure 5E,F). RSAE and nimodipine are similar in their ability to regulate the concentrations of MDA and NO and the activity of SOD (*p* < 0.01, Figure 5D–F, respectively).

### 2.5. Representative Images of HE Staining and Nissl Staining

**Figure 6:**
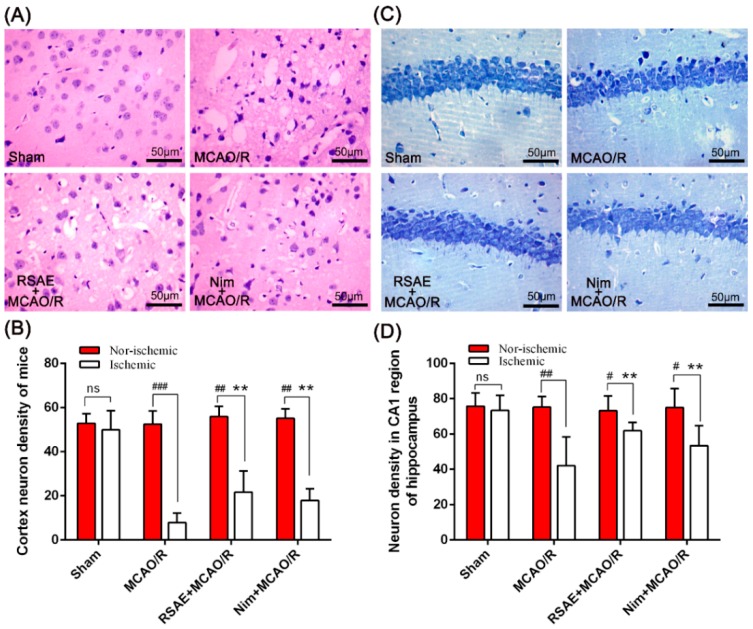
Representative images of H&E and Nissl staining performed on sections from ischemic brain at 24 h after ischemia/reperfusion in mice. (**A**) Representative H&E staining in the cortex at 24 h after ischemia/reperfusion. (**B**) The ischemic and nonischemic cortex neuron density of mice after focal cerebral ischemia. (**C**) Representative Nissl staining in the cortex at 24 h after ischemia/reperfusion. (**D**) The neuron density in the CA1 region of the hippocampus of mice after focal cerebral ischemia. Mean values ± standard error of the mean (n = 10); * *p* < 0.05, ** *p* < 0.01 versus MCAO/R group; ^#^ *p* < 0.05, ^##^ *p* < 0.01, ^####^ *p* < 0.0001 versus sham-operated group; ns means not significant. Scale bar, 50 μm.

The neuroprotective effects of RSAE on cerebral ischemia and reperfusion, which normally leads to neurological damage and morphological changes, was confirmed by Nissl and H&E staining of sections from the ischemic cortex at 24 h following ischemia/reperfusion in mice (Figure 6).

As shown in Figure 6A,C, nerve cells of the sham operation group presented a normal structural form: the cell outline was clear, the structure was compact, and the nucleolus was clearly visible, similar to the nonischemic brain tissue of the MCAO/R model group and of the RSAE-treated group.

However, edema and necrosis were observed in the ischemic cortex and hippocampus of the MCAO/R group (Figure 6A): the gap around some neurons was enlarged, the nerve cells were sparse, the neurons were swollen or shrunk, the nucleolus disappeared, the nuclear membrane was dissolved, and the nucleus was solid. Compared with the model group, the ischemic changes in nerve cells were alleviated by RSAE pretreatment: only some nerve cells were degenerated and necrotic, the cell body deformation was reduced, and the number of viable cells was greater than that of the model group.

Compared with the sham operation, after focal cerebral ischemia, the ischemic cortex neuron density significantly decreased from 49.93 ± 8.71 to 7.87 ± 4.27 (*p* < 0.001, Figure 6B), and the focal cerebral ischemia (MCAO/R) operation significantly decreased ischemic neuron density in the CA1 region of the hippocampus from 73.38 ± 8.51 to 42.13 ± 16.21 (*p* < 0.01, Figure 6D). However, RSAE pretreatment (2.4 g·kg^−1^ of the original drug) substantially reduced neuronal damage from MCAO/R and significantly increased the ischemic cortex neuron density to 21.70 ± 9.57 (*p* < 0.01, Figure 6B), and the ischemic neuron density in the hippocampus CA1 region significantly increased to 61.90 ± 4.75 (*p* < 0.01, Figure 6D).

### 2.6. Expression of Bax and Bcl-2 in Mice with Focal Cerebral Ischemia

**Figure 7:**
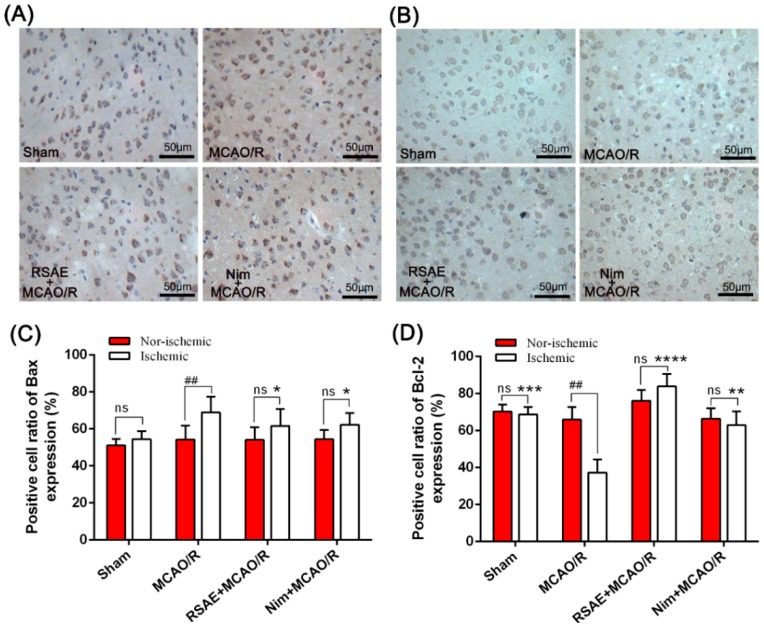
Effect of RSAE on the levels of Bax- and Bcl-2-positive cells in mice with focal cerebral ischemia at 24 h after ischemia/reperfusion. (**A**) Representative immunohistochemical staining images of pro-apoptotic Bax in the ischemic and nonischemic neuron sections at 24 h after ischemia/reperfusion. (**B**) Representative immunohistochemical staining images of anti-apoptotic Bax in the ischemic and nonischemic neuron sections at 24 h after ischemia/reperfusion. (**C**) Quantitative analysis of Bax-positive cells in mice with focal cerebral ischemia at 24 h after ischemia/reperfusion. (**D**) Quantitative analysis of Bcl-2-positive cells in mice with focal cerebral ischemia at 24 h after ischemia/reperfusion. Mean values ± standard error of the mean (n = 10); * *p* < 0.05, ** *p* < 0.01, ***** *p* < 0.0001 versus MCAO/R group; ^#^ *p* < 0.05, ^##^ *p* < 0.01, ^####^ *p* < 0.0001 versus sham-operated group; ns means not significant. Scale bar, 50 μm.

To further investigate the effects of RSAE on MCAO/R-induced brain tissue apoptosis, immunohistochemistry was used to detect Bax (pro-apoptotic protein) and Bcl-2 (anti-apoptotic protein) expression levels in the ischemic and nonischemic cortex and in the hippocampus region. It was found that positive cells were yellow in the cytoplasm or nucleus. Three sections of ischemic brain tissue with 10 × 40 magnification (400×) were randomly selected for image analysis. As shown in Figure 7A,B, the positive expression of Bax and Bcl-2 was characterized by deep brown-yellow staining of the cytoplasm and concentrated staining around the infarct.

As displayed in Figure 7D, compared to the sham group, the ratio of Bax-positive cells in the ischemic peripheral area of the middle cerebral artery occlusion/reperfusion (MCAO/R) model group was significantly increased from 54.47 ± 4.34 to 68.93 ± 8.54 after focal cerebral ischemia in the mice (*p* < 0.01). However, compared with the model group, the expression of Bax in the ischemic peripheral area was significantly reduced to 61.63 ± 9.10 by RSAE pretreatment (2.4 g·kg^−1^ of the original drug) (*p* < 0.01, Figure 7C). In contrast, Bcl-2 expression (the ratio of Bcl-2-positive cells) in the MCAO/R model group significantly decreased from 68.73 ± 1.95 to 37.30 ± 3.15 compared to that in the control group (*p* < 0.001, Figure 7D). In addition, RSAE significantly increased the ratio of Bcl-2-positive cells to 83.87 ± 2.05 compared to that in the MCAO/R model group (*p* < 0.0001, Figure 7D). Moreover, the ratio of Bax- and Bcl-2-positive cells presented no significant difference between ischemic and nonischemic brain neuron sections of mice after focal cerebral ischemia, excluding the MCAO/R model group.

### 2.7. ERK1/2, JNK1/2 and p38 MAPK in Mice after MCAO/R

It is known that the activation of MAPKs signaling pathways plays a critical protective role against OGD/R and MCAO/R-induced apoptosis and could regulate the phosphorylation of ERK1/2 JNK1/2 and p38 MAPK to exert neuroprotective effects [8,9,10,11,13,25,26,27]. Based on the effects of RSAE on oxidative stress in OGD/R and MCAO/R models, the association between oxidative stress damage induced by OGD/R and MCAO/R models and the subsequent activation of MAPK was elucidated. 

**Figure 8:**
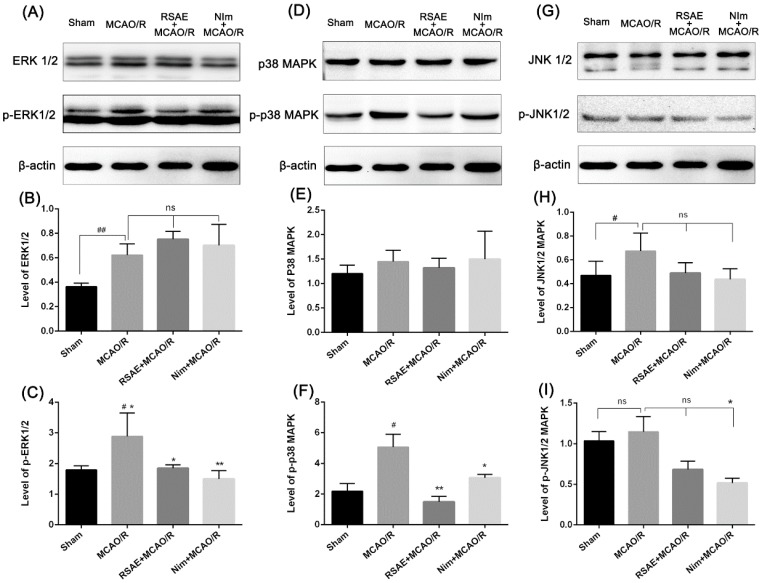
Effects of RSAE on the expression of phosphorylated ERK1/2, JNK1/2, and P38 in the hippocampus of ischemic brain sections. (**A**,**D**,**G**) The protein levels of phospho-ERK, phospho-P38, and phospho-JNK, respectively, in the hippocampus of ischemic brain sections were examined by western blot analysis. (**B**,**C**,**E**,**F**,**H**,**I**) The IOD values of ERK1/2, p-ERK, JNK1/2, p-JNK1/2, P38 and p-P38, respectively, were quantified and analysed using Gel-Pro analyzer software. Mean values ± standard error of the mean (n = 3); * *p* <0.05, ** *p* < 0.01, versus MCAO/R group; ^#^ *p* < 0.05, ^##^ *p* < 0.01 versus sham-operated group; ns means not significant.

As shown in Figure 8A,B, the protein levels of ERK1/2 and phosphorylated ERK (p-ERK) in mice with focal cerebral ischemia were significantly increased in the MCAO/R model (*p* < 0.01 and *p* < 0.05, respectively). Comparatively, pretreatment with RSAE reduced the phosphorylation levels of ERK (p-ERK) and did not significantly affect the protein levels of ERK1/2, indicating that RSAE may regulate the phosphorylation levels of ERK to produce a cerebral anti-ischemic effect. Similarly, the increased phosphorylation of P38 induced by MCAO/R was strikingly abrogated by pretreatment with RSAE (*p* < 0.01, Figure 8D,F), indicating that RSAE may also regulate the phosphorylation levels of ERK to produce a cerebral anti-ischemic effect. Moreover, RSAE did not significantly affect JNK1/2 and its phosphorylation levels (Figure 8G–I). These results indicated that RSAE may play a key protective role against focal cerebral ischemia in mice by suppressing the phosphorylation levels of ERK1/2 and p38 MAPK.

## 3. Discussion

It has been reported that in ischemic stroke, energy metabolism disorders and mitochondrial dysfunction may lead to the formation of large amounts of free radicals, mediate oxidative damage to DNA, inhibit antioxidant enzyme activity, upregulate Bax levels, downregulate Bcl-2 levels in astrocytes, and finally, suppress the activation of Caspase-3, thus inducing neuronal apoptosis [28]. Our research found that RSAE could increase the antioxidant capacity to mediate oxidative stress in OGD/R-induced PC12 cells and MCAO/R model mice: SOD, CAT and GSH-Px activity was remarkably improved (Figure 3 and Figure 5), indicating that ROS may be reduced as a result of inhibition of MCAO/R-induced ROS production, which leads to mitochondrial damage and induces mitochondrial apoptotic pathways. RSAE can reduce ROS accumulation caused by mitochondrial damage and energy metabolism disorders and inhibit neuronal cell injuries and apoptosis induced by oxidative stress (Figure 2 and Figure 3). Therefore, our results showed that pretreatment with RSAE (12.5 μg/mL) for 8 h or 16 h significantly improved cell viability (Figure 2) and inhibited OGD/R-induced damage (Figure 3), indicating that RSAE may inhibit activated oxidative stress, reduce ROS production and decrease the mitochondrial membrane potential (MMP) (Figure 4) to exert neuroprotective effects.

As is known, activation of apoptosis can be induced by growth factor deficiency, toxins, and glucose-oxygen deficiency. It has also been found that apoptosis can be controlled by a variety of regulators and regulatory proteins that possess inhibitory (anti-apoptosis) effects on programmed cell death or block (pro-apoptosis) inhibitors. To prevent target cells from activating apoptosis too soon, each cell encodes their own anti-apoptosis genes as a defensive strategy [29]. Bcl-2 and Bax are two major members of the Bcl-2 family, a family of proteins that primarily regulates cell apoptosis and necrosis.

Bax is a cytosolic sensor of cell damage and stimulation that functions by forming pores across the mitochondrial outer membrane, leading to a decrease in the MMP along with an outflow of Cyt C and other apoptosis-inducing factors. Bax also activates Caspase-3 and destroys the anti-apoptotic function of the Bcl-2 protein under normal conditions [30]. The anti-apoptotic protein Bcl-2 is an important intracellular component that inhibits the overexpression of Bax by blocking Cyt C release from mitochondria and Caspase-3 aggregation and that links the mitochondrial outer membrane to stabilize membrane permeability and protect mitochondrial integrity, which, in turn, inhibits apoptosis [30]. Both Bax and Bcl-2 play an important regulatory role in the mitochondrial pathway; therefore, the ratio of Bax/Bcl-2 is considered an important indicator of apoptosis. 

In this study, we used immunohistochemistry to detect changes in the expression of Bax and Bcl-2. It was suggested that the anti-apoptotic protection effects of RSAE treatment on ischemic stroke resulted from upregulating Bcl-2 protein expression and downregulating Bax protein expression (Figure 4 and Figure 6). 

Thus, we speculated that since RSAE exerts an anti-apoptotic effect, it may inhibit the activation of Caspase-3 by regulating the expression of Bcl-2 and Bax. Concurrently, RSAE pretreatment increased antioxidant activity and stabilized the mitochondrial membrane potential, indicating that it may exert a protective effect by regulating oxidative stress and activation of apoptosis [8,9,10,13,18,27,28].

Throughout the course of ischemic stroke, cerebral I/R following a cerebral infarction can cause neuronal apoptosis, reaching the summit 1 to 5 days after ischemia, and can last for approximately 4 weeks [4,14,31,32]. One study revealed that apoptosis was a dynamic progressive process that follows cerebral ischemia-reperfusion. During the early stage at 30 min, edema appeared in the anterior synovial striatum ischemic area surrounded by the appearance of a minor number of apoptotic cells, and with the prolongation of reperfusion time (6–72 h), the number of apoptotic cells increased. These cells were mainly located in the penumbra of the brain slices. The CA1 area of the hippocampus is considered to be sensitive to ischemic injury. After a certain time, ischemia-induced damage cannot be suppressed or corrected; consequently, the penumbra in the ischemic brain region may inevitably deteriorate and become part of a permanent infarct [32]. In the central area of cerebral ischemia, the cells were mainly necrotic, with cell death primarily caused by apoptosis around the ischemic area [8,9,10]. Similarly, our research showed that the number of positive cells in the ischemic (MCAO/R) group was significantly higher than that in the sham-operated group and that the number of neuron-positive cells was significantly decreased (Figure 7). While the number of apoptosis-positive cells in the RSAE-treated group was significantly lower than that in the MCAO/R group, the number of positive cells increased. Furthermore, the infarct size of the intervention group was significantly smaller than that of the ischemic group, which indicated that RSAE seemed to exert neuroprotective effects on cerebral ischemia and reperfusion injury by inhibiting apoptosis.

Based on the present reports, the precise mechanism of neuronal death resulting from ischemic injury was not completely elucidated; this process is dependent on the severity of the injury and quantity of apoptotic cells, in addition to the mechanism of occurrence, location, extent, and duration of ischemia. Moreover, a timely and effective method of neuronal apoptosis inhibition during cerebral ischemia has not yet been fully resolved, but a large number of studies have shown that neuronal apoptosis is related to cell cycle regulation [13,18,27,28].

In our research, western blot analysis showed that MCAO/R significantly increased the phosphorylation levels of ERK1/2 and p38 MAPK proteins in the ischemic penumbra compared to those of controls. However, these increased phosphorylation levels of ERK1/2 and p38 induced by MCAO/R were strikingly abrogated by pretreatment with RSAE, which indicated that RSAE may modulate the ERK1/2 and p-38 MAPK pathway by mediating the phosphorylation levels of MAPK pathway components in MCAO/R mice. In addition, RSAE had no effect on p-JNK1/2, JNK1/2 and p38 expression (Figure 8), indicating that RSAE may regulate cytokine expression and suppress cell apoptosis after stroke at least partially by modulating the MAPK pathway. However, it has not yet been determined whether RSAE binds directly to membrane receptors or regulates MAPKs through a secondary signal. Furthermore, the identification of specific active ingredients in RSAE has not yet been performed, necessitating further in-depth studies.

## 4. Methods

### 4.1. RSAE Preparation

Radix Scrophulariae was purchased from Xinyi Town, Chun’an Town, Zhejiang Province in China; the aqueous extract of Radix Scrophulariae (RSAE) was prepared by the Chinese Herbal Medicine Germplasm Resources and Evaluation Laboratory of Zhejiang University of Traditional Chinese Medicine, Hangzhou, in China. According to experimental requirements, RSAE was prepared and diluted to different concentrations.

### 4.2. Cell Cultures and OGD/R Models

PC12 (pheochromocytoma) cells were obtained from the Institute of Basic Medical Sciences at the Chinese Academy of Medical Sciences [33] and were cultured at 37 °C in Dulbecco’s modified Eagle’s medium (Gibco, Grand Island, NY, USA) with 10% foetal bovine serum (Gibco, Grand Island, NY, USA), 100 U/mL penicillin and 100 mg/mL streptomycin in a normal incubator, which was used as complete medium (CM) containing 4.5 g/L D-glucose. PC12 cells at a density of 1 × 10^5^ cells/mL were seeded in 96-well plates in a total volume of 100 μL per well, and the cells were allowed to adhere and grow for 24 h. 

According to the oxygen-glucose deprivation and reperfusion (OGD/R) model established in our lab [33,34], we made proper adjustments, replaced growth medium with glucose-free DMEM (Gibco, U.S.), and placed the plates into an anaerobic incubator under 95% N_2_ and 5% CO_2_ at 37 °C for 120 min. Then, the glucose-free DMEM was rapidly replaced with CM so that cells were fed for a 24 h reperfusion period with complete medium in a normal incubator. Control cell cultures were incubated under normal conditions and were not deprived of oxygen or glucose. The cells were pretreated with different concentrations of RSAE, namely, 6.25 μg/mL, 12.5 μg/mL, 25.0 μg/mL and 50.0 μg/mL, and were then exposed to OGD/R injury.

### 4.3. Cell Viability Analysis

Cell viability of PC12 cells was assessed according to the MTT assay. After OGD/R, the 3-[4,5-dimethylthiazol-2-yl]-2,5-diphenyltetrazolium bromide (Amresco, Atlanta, GA, USA) assay was used to detect cell viability as previously described [33,34,35]. Briefly, MTT was dissolved in DMEM and added to each well for incubation at a final concentration of 0.5 mg/ml at 37 °C for 4 h. Then, to dissolve the formazan crystals, the medium was replaced with 150 μL of DMSO per well for 10 min. An automatic microplate reader (Spectrauor, TECAN, Sunrise, Austria) was used to record the optical density (OD) of all groups at 570 nm. Cell viability was expressed as a percentage of the control value. Each experiment was repeated in quintuplicate using three independent cultures.

### 4.4. Assessment of Cell Injury or Death

Cell viability analysis showed pretreatment with 12.5 μg/mL RSAE could improve the viability of OGD/R-induced PC12 cells. To confirm the reliability of the MTT results, we used diagnostic kits to measure the amount of LDH leakage release as an indicator of cell injury or death. The four sets of experiments were carried out as follows: (1) control PC12 cells, (2) OGD/R-induced PC12 cells, (3) PC12 cells pretreated with 12.5 μg/mL RSAE for 24 h and then induced by OGD/R, and (4) control PC12 cells pretreated with 12.5 μg/mL RSAE.

Based on the manufacturer’s instructions, the amount of LDH leakage release was determined using an assay kit (Nanjing Jiancheng Bioengineering Institute, Nanjing, China). Culture media were harvested after OGD/R injury to determine LDH levels. LDH leakage was expressed as a percentage of total LDH activity (LDH in the medium + LDH in the cells).

### 4.5. Determination of the Levels of Oxidative Stress

Following treatment, PC12 cells induced by OGD/R were lysed using 0.2% Triton X-100 for 30 min to release intracellular superoxide malondialdehyde (MDA), dismutase (SOD), glutathione peroxidase (GSH-Px) and catalase (CAT). The levels of oxidative stress were determined by quantifying MDA, SOD, GSH-Px and CAT enzyme activity using an automatic microplate reader (Spectrauor, TECAN, Sunrise, Austria) according to the kit instructions [31]. Additionally, a BCA Protein Assay Kit (Nanjing Jiancheng Bioengineering Institute, Nanjing, China) was used to detect protein concentrations according to the manufacturer’s instructions. The following four sets of experiments were performed: (1) control PC12 cells, (2) OGD/R-induced PC12 cells, (3) cells pretreated with 12.5 μg/mL RSAE for 24 h and then induced by OGD/R, and (4) control cells pretreated with 12.5 μg/mL RSAE.

### 4.6. Measurement of the Mitochondrial Membrane Potential

The change in the mitochondrial membrane potential (MMP) was detected by JC-1 staining [36]. PC12 cells (cell density, 1 × 10^5^ cells/mL) were cultured in 24-well plates. Posttreatment, cells were harvested and incubated with JC-1 (2 mM final concentration) at 37 °C in the dark for 30 min. Then, the cells were immediately observed using fluorescence microscopy (Leica, Germany Q9). The results were analysed using the ImageJ software (National Institutes of Health, Bethesda, MD, USA) [33]. The following four sets of experiments were performed: (1) control PC12 cells, (2) OGD/R induced-PC12 cells, (3) cells pretreated with 12.5 μg/mL RSAE for 24 h and then induced by OGD/R, and (4) control cells pretreated with 12.5 μg/mL RSAE.

### 4.7. Mouse MCAO/R Model and Drug Administration

Male Kunming (KM) mice (SPF, weighed 18–22 g) used in this study were purchased from the Animal Experimental Center of Zhejiang University of Traditional Chinese Medicine. Mice were housed at an ambient temperature of 20 ± 1 °C with a 12-h light/dark cycle and free access to a standard laboratory chow diet and water. All human subjects gave informed consent for inclusion before participation in the study. The study was conducted in accordance with the Declaration of Helsinki. The protocol was approved by the Laboratory Animal Ethics Committee of the Institute of Medicinal Plant Development, Peking Union Medical College, and conformed to the Guide for the Care and Use of Laboratory Animals (Permit Number: SYXK 2017-0020).

For focal brain ischemia, the mouse middle cerebral artery occlusion and reperfusion model (MCAO/R) was used as described previously [37,38,39,40]. In brief, animals were anaesthetized with 50 mg·kg^−1^ Zoletil 50 via intramuscular injection (Virbac S.A, Carros, France) and maintained at a half-dose. A silicone-coated 8–0 monofilament was introduced into the left internal carotid artery and advanced to occlude the middle cerebral artery for 2 h. After 2 h MCAO, the animals were briefly re-anaesthetized, and the filament was withdrawn for reperfusion studies.

All animals were divided into 4 groups (n = 10 per group) according to the random number table, namely, the MCAO/R operation model group; the sham operation group; the group treated with 2.4 g·kg^−1^ Radix Scrophulariae aqueous extract (RSAE), a dose equivalent to the original drug; and the positive drug group, with a dose of 12 mg·kg^−1^ nimodipine. Continuous gastric administration lasted for 7 days, once per day. The model and sham operation groups were given an equal volume of physiological saline for injection.

### 4.8. Quantification of Infarct Volume and Brain Water Content

After the MCAO/R operation, mice were anaesthetized with 50 mg·kg^−1^ Zoletil 50 via intramuscular injection (Virbac S.A, Carros, France), and brains were removed, sectioned coronally at a thickness of 2 mm, incubated in a phosphate-buffered solution (pH = 7.3) with 2% (*w*/*v*) 2,3,5-triphenyltetrazolium chloride (TTC), and stained for 15 min at 37 °C, followed by overnight immersion [41]. The normal nonischemic tissue was stained red, while the infarct tissue area remained unstained (white). The infarct areas on each slice were quantified by using ImageJ software. To compensate for the effect of brain edema, the corrected infarct volume was calculated as described previously (ten male mice per group): Corrected infarct area = left hemisphere area − (right hemisphere area-infarct area) [42].

The mice were anaesthetized, and mouse brain tissue was acquired and processed as needed at 24 h after MCAO. A 3-mm section of the ischemic hemisphere brain was cut from the anterior pole to detect the water content in the brain tissue. The wet-dry method was applied to determine brain water content (BWC) in another subgroup (n = 10 per group). An electronic scale was used to weigh the ischemic and nonischemic hemispheres (wet weight). After the ischemic brain hemisphere was dried overnight at 105 °C in a desiccating oven, it was weighed again (dry weight), and the total brain water content was calculated according to BWC % = [(wet weight − dry weight)/wet weight] × 100% [43,44].

### 4.9. Assessment of the Levels of Superoxide Dismutase Activity and Malondialdehyde and Nitric Oxide Contents

At 24 h post-surgery, animals were sacrificed and decapitated, and the right hemisphere of the brain was retrieved. The remaining brain tissue was washed with prechilled saline before blood was removed, and the tissue was then dried on filter paper. After being weighed, tissues were prepared as 10% brain tissue homogenate in a homogenizer and centrifuged at 3000 rpm·min^−1^ for 10 min. The supernatant was collected and stored at −20 °C. The Bradford method was used to determine protein concentrations in the collected supernatants. SOD activity was determined by xanthine oxidase, the MDA content was determined by thiobarbituric acid, and the nitric oxide (NO) content was determined by nitrate reductase. All the determinations were carried out using assay kits (Nanjing Jiancheng Bioengineering Institute, Nanjing, China) [31]. Ten male mice were used for each group.

### 4.10. Haematoxylin-Eosin and Nissl Staining

Following the MCAO/R operation, animals were anaesthetized with Zoletil 50 and immediately sacrificed. Then, the animals were perfused through the heart with 4°C phosphate-buffered saline (PBS), followed by 4% paraformaldehyde. Finally, the intact brain was carefully removed and placed in brain buffer containing 4% formaldehyde fixative for haematoxylin and eosin staining (H&E) staining, toluidine blue staining and other immunohistochemical staining.

Haematoxylin-eosin (H&E) staining was used to revealed morphological features of injured neurons in the cerebral cortex. The samples were embedded in paraffin and cut in 5-μm slices; 5-μm-thick serial coronal sections were generated and mounted on slides. The sections were stained using haematoxylin and H&E according to the described standard protocol [39,45]. Images of stained slides were acquired using a light microscope (Leica, Wetzlar, Germany). Nissl staining was applied to observe morphologic changes in hippocampus cells within the ischemic penumbra after MCAO/R operation, mainly containing CA1 in the hippocampus [44]. After 24 h following MCAO/R operation, the brains were processed as above. Samples were embedded in paraffin and cut in 5-μm slices; 5-μm-thick serial coronal sections were generated and mounted on slides. After the sliced sections were washed in cold water, the paraffin sections were stained with 1% toluidine blue for 20 min, rinsed with PBS, dehydrated with graded alcohol, made transparent with xylene, and fixed with neutral glue. An optical microscope was used to observe each section.

Normal neurons showed typical cell size, prominent protrusions, uniform cytoplasm, a clear nuclear membrane, obvious nucleoli, and no chromatin condensation. Three high-magnitude (10 × 40) fields in both the cortex and the CA1 of the hippocampus were randomly selected for image analysis; the number of pyramidal cells was also counted. The mean value was taken as the number of pyramidal cells in the sample (n = 10 per group).

### 4.11. Immunohistochemical Evaluation

After MCAO/R operation, the animals were anaesthetized with Zoletil 50 and immediately sacrificed. The tissue samples obtained from mice were histologically processed and embedded in paraffin for immunohistochemical staining. Five micro slides were removed from these blocks and mounted on poly-L-lysine coated slides; these slides were immunohistochemically stained with primary antibodies recognizing Bcl-2 and Bax. The remaining procedure was performed as described previously with little modification [12,16]. Finally, the slides were fixed with a water-based mounting medium and evaluated under an optical microscope. Three sections of ischemic brain tissue with 10 × 40 magnification (400×) were randomly selected for image analysis via ImageJ software.

### 4.12. Western Blot Analysis

Twenty-four hours after MCAO, ischemic brain sections that included both the ischemic area and corresponding regions from the contralateral hemisphere were collected, and protein extracts were prepared as described previously [33,36]. PVDF membranes (Millipore, Bedford, MA, USA) were blocked for 2 h in 5% nonfat milk in Tris-buffered saline (TBS)/Tween 20 and probed with the following antibodies: anti-Bax (1:200), anti-Bcl-2 (1:200), anti-p-p38 (1:500), anti-p-38 (1:500), anti-p-ERK (1:200), anti-ERK (1:200), anti-cleaved caspase-3 (1:500), anti-p-JNK (1:200), anti-JNK (1:200), and anti-β-actin (1:1000). Protein expression was detected by an enhanced chemiluminescence method and imaged by using ChemiDoc XRS (Bio-Rad, Hercules, CA, USA). To reduce variations in protein expression quantification, three independent experiments were performed. 

### 4.13. Statistical Analysis

Data are presented as the mean values ± standard error of the mean. All analyses were performed using GraphPad Prism 7.0 statistical software (GraphPad Software, Inc., La Jolla, San Diego, CA, USA). One-way ANOVA was used to determine the differences between groups, and then post-hoc LSD testing was applied. Comparisons between two groups were performed by using unpaired Student’s t-test. The *p* values < 0.05 were considered significant. At least three independent experiments were performed for each experiment.

## 5. Conclusions

**Figure 9:**
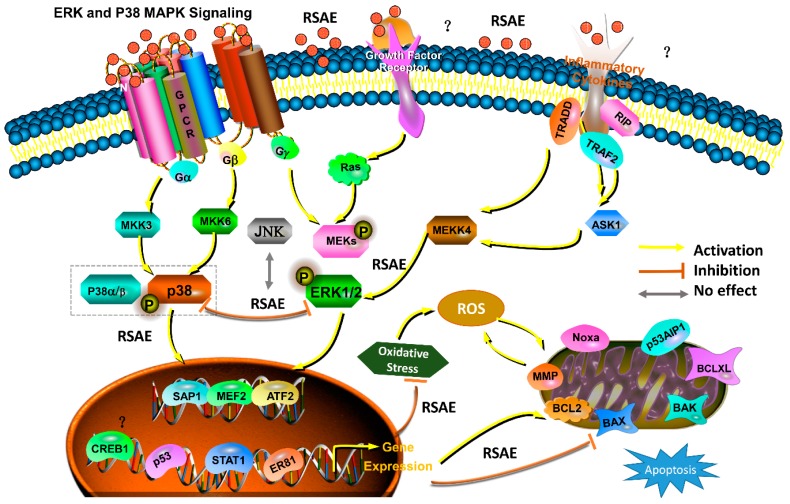
Graphic summary of the neuroprotective effects that Radix Scrophulariae aqueous extract exerts on cerebral ischemia and reperfusion injury by inhibiting apoptosis via the ERK1/2 and p-38 MAPK pathways.

In summary, as shown in Figure 9, our research initially proved that Radix Scrophulariae aqueous extract (RSAE) exerts neuroprotective effects on cerebral ischemia and reperfusion (I/R) injury in oxygen-glucose deprivation and reperfusion (OGD/R)-induced PC12 cells and in middle cerebral artery occlusion/reperfusion (MCAO/R) model mice. These neuroprotective effects were associated with attenuated infarct volume, brain water content, and nitric oxide (NO) and malondialdehyde (MDA) concentrations. Collectively, these effects inhibit I/R-induced damage by reducing the levels of LDH leakage release; improving antioxidant capacity by upregulating SOD, GSH-Px and CAT activity; stabilizing the mitochondrial membrane potential; reducing neuronal apoptosis, necrosis, and loss of neurons by regulating the expression of the anti-apoptotic and pro-apoptotic proteins Bcl-2 and Bax, respectively; and downregulating the phosphorylation levels of MAPK pathway components. Our findings highlight the neuroprotective effects of RSAE on cerebral I/R injury in mice and their potential mechanism.

Since Radix Scrophulariae is already regarded as a potential neuroprotective natural plant, our results may offer directions and clues for discovering novel active compounds or drugs for the treatment of ischemic stroke by chemically separating and identifying active compounds from this extract. Furthermore, this work may provide new insights into therapeutic targets for stroke patients.
