# Correction: Meng, X., et al. Neuroprotective Effects of Radix Scrophulariae on Cerebral Ischemia and Reperfusion Injury via MAPK Pathways. *Molecules* 2018, 23, 2401

**DOI:** 10.3390/molecules23102704

**Published:** 2018-10-19

**Authors:** Xiangbao Meng, Weijie Xie, Quanfu Xu, Tian Liang, Xudong Xu, Guibo Sun, Xiaobo Sun

**Affiliations:** 1 Beijing Key Laboratory of Innovative Drug Discovery of Traditional Chinese Medicine (Natural Medicine) and Translational Medicine, Institute of Medicinal Plant Development, Peking Union Medical College and Chinese Academy of Medical Sciences, Beijing 100193, China; xbmeng@implad.ac.cn (X.M.); ginseng123@163.com (W.X.); shengjupan@163.com (Q.X.); liantianparper@sina.com (T.L.); 2 Key Laboratory of Bioactive Substances and Resource Utilization of Chinese Herbal Medicine, Ministry of Education, Beijing 100193, China; 3 Key Laboratory of Efficacy Evaluation of Chinese Medicine against Glycolipid Metabolic Disorders, State Administration of Traditional Chinese Medicine, Beijing 100193, China; 4 Zhongguancun Open Laboratory of the Research and Development of Natural Medicine and Health Products, Beijing 100193, China

The authors wish to make the following correction to their paper [1]:

We found that in the Authorship section, below the title, a mistake exists on the published page. The first author name, which is stated as “Xiaobo Meng”, should be corrected to “Xiangbao Meng”. And in the Abstract section and at the 43th line, “Ischemia and reperfusion-induced injury of or apoptosis inhibition” should be corrected to “Ischemia and reperfusion-induced injury or apoptosis inhibition”.

The changes do not affect the scientific results. The manuscript will be updated and the original will remain online on the article webpage, with a reference to this correction.
